# Um Modelo Preditivo para Embolia Pulmonar em Pacientes com Fibrilação Atrial Baseado em Tromboelastografia e Parâmetros de Coagulação

**DOI:** 10.36660/abc.20250683

**Published:** 2026-08-24

**Authors:** Xingqi Huang, Jianmei Liu

**Affiliations:** 1 Department of Blood Transfusion Jinhua Guangfu Oncology Hospital Jinhua China Department of Blood Transfusion, Jinhua Guangfu Oncology Hospital, Jinhua – China; 2 Department of Cardiovascular Medicine Jinhua Guangfu Oncology Hospital Jinhua China Department of Cardiovascular Medicine, Jinhua Guangfu Oncology Hospital, Jinhua – China

**Keywords:** Fibrilação Atrial, Embolia Pulmonar, Tromboelastografia

## Abstract

**Fundamento:**

Pacientes com fibrilação atrial (FA) apresentam risco elevado de embolia pulmonar (EP), porém as ferramentas de predição existentes demonstram precisão limitada.

**Objetivos:**

Este estudo teve como objetivo desenvolver e validar um novo modelo preditivo que integra parâmetros de tromboelastografia (TEG) com marcadores de coagulação convencionais para avaliação do risco de EP em pacientes com FA.

**Métodos:**

Realizamos um estudo retrospectivo com 271 pacientes hospitalizados com FA submetidos a angiotomografia computadorizada pulmonar (angioTC) para investigação de EP (41 com EP, 230 sem). A idade média foi de 76,5 anos, 56,5% eram do sexo masculino e a hipertensão foi a comorbidade mais comum (67,5%). O diagnóstico de EP foi confirmado por angiotomografia computadorizada pulmonar. Foram analisadas as características basais, os parâmetros da TEG (incluindo tempo de reação, amplitude máxima [AM] e ângulo α) e os marcadores de coagulação padrão (dímero-D, fibrinogênio). A significância estatística foi definida como um valor de p bicaudal < 0,05. A regressão logística multivariada identificou preditores independentes, que foram incorporados a um nomograma. O desempenho do modelo foi avaliado por meio de análise da curva ROC (característica de operação do receptor), curvas de calibração e análise da curva de decisão (ACD).

**Resultados:**

A prevalência de EP foi de 15,1%. Pacientes com EP apresentaram níveis significativamente mais elevados de TEG-AM, dímero-D e fibrinogênio em comparação ao grupo sem EP (todos com p < 0,05). A análise multivariada identificou esses três marcadores como preditores independentes. O nomograma demonstrou excelente discriminação (AUC = 0,878, IC 95%: 0,806-0,949), com sensibilidade de 87,8% e especificidade de 78,0% no ponto de corte ideal. O modelo apresentou boa calibração (p de Hosmer-Lemeshow = 0,965) e significativa utilidade clínica.

**Conclusão:**

O nomograma baseado em TEG, que combina AM, dímero-D e fibrinogênio, fornece uma estratificação de risco de EP precisa e acessível à beira do leito para pacientes com FA. Essa ferramenta pode facilitar a identificação precoce de indivíduos de alto risco e orientar a tomada de decisões clínicas em relação à terapia anticoagulante. Estudos prospectivos adicionais são necessários para validar esses achados.

## Graphical abstract

**Figura Central:**
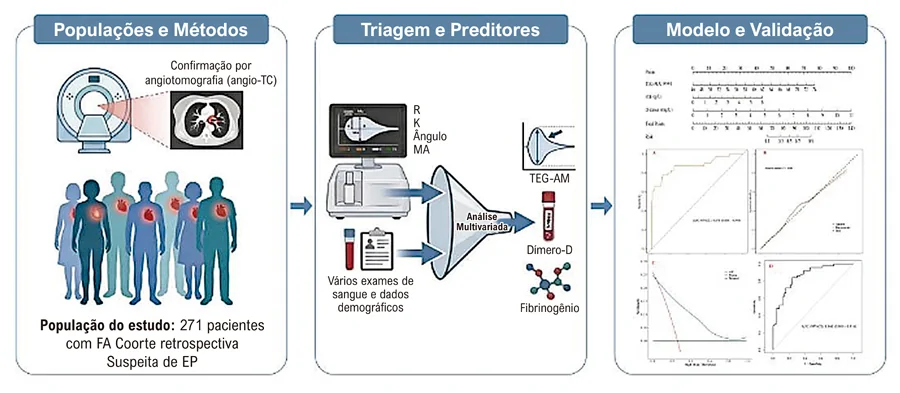
Um Modelo Preditivo para Embolia Pulmonar em Pacientes com Fibrilação Atrial Baseado em Tromboelastografia e Parâmetros de Coagulação

## Introdução

A fibrilação atrial (FA), a arritmia cardíaca sustentada mais comum na prática clínica, está se tornando cada vez mais prevalente com o envelhecimento da população mundial e emergiu como uma importante preocupação de saúde pública. Diretrizes recentes da Sociedade Europeia de Cardiologia e da *American Heart Association/American College of Cardiology* enfatizam a importância da avaliação das complicações associadas à FA.^1,2^A embolia pulmonar (EP) é uma complicação grave, porém frequentemente subdiagnosticada, em pacientes com FA. Estudos demonstraram que a incidência de EP foi de 1,55 por 1.000 pessoas-ano em pacientes com FA, representando um risco substancialmente maior em comparação com aqueles sem FA.^3^A coexistência de EP prolonga significativamente a hospitalização e aumenta a mortalidade, especialmente entre pacientes idosos e aqueles com múltiplas comorbidades, levando a desfechos acentuadamente piores a curto e longo prazo.^4,5^Além disso, as manifestações clínicas inespecíficas tanto da FA quanto da EP — como dispneia e palpitações — podem facilmente levar a diagnósticos incorretos ou perdidos, exacerbando, assim, a carga da doença e o consumo de recursos de saúde.^6^

Atualmente, o diagnóstico de EP em pacientes com FA permanece um desafio clínico. O diagnóstico definitivo de EP baseia-se principalmente em exames de imagem, sendo a angiotomografia computadorizada pulmonar (angioTC) considerada o padrão ouro. Embora a angioTC tenha alto valor diagnóstico, ela também apresenta limitações, como exposição à radiação ionizante, risco de nefropatia induzida por contraste e potencial sobrediagnóstico de êmbolos subsegmentares.^7^Dentre os marcadores laboratoriais, o dímero-D é comumente utilizado como triagem auxiliar devido à sua alta sensibilidade. No entanto, sua especificidade é baixa e é facilmente influenciada por fatores como idade, comorbidades e estados inflamatórios, o que pode levar a um aumento nas taxas de falsos positivos.^8^Sistemas de pontuação clínica, como os escores de Wells e de Genebra, são úteis na população em geral, mas seu desempenho pode ser reduzido em pacientes hospitalizados complexos com sintomas cardiopulmonares sobrepostos e múltiplas comorbidades.^9,10^Essas limitações ressaltam a necessidade de biomarcadores mais confiáveis, dinâmicos e reproduzíveis para auxiliar na identificação precoce do risco de EP em pacientes com FA, melhorando, em última análise, a tomada de decisões clínicas e o manejo prognóstico.

A tromboelastografia (TEG) é um ensaio dinâmico que avalia todo o processo de coagulação, fornecendo informações abrangentes sobre coagulação, fibrinólise e função plaquetária.^11,12^Ao contrário dos testes de coagulação convencionais, a TEG permite o monitoramento em tempo real do estado de coagulação do paciente e tem sido amplamente utilizado para avaliação do risco de sangramento e trombose em contextos de trauma, oncologia e terapia intensiva.^13-15^Nos últimos anos, estudos preliminares exploraram a utilidade da TEG na previsão de tromboembolismo venoso (TEV), sugerindo seu potencial para identificação precoce.^16^No entanto, as evidências sobre a aplicação da TEG na FA — uma população com risco trombótico particularmente elevado — ainda são limitadas. Portanto, a construção de um modelo preditivo baseado em parâmetros da TEG, em combinação com índices laboratoriais convencionais, pode ajudar a preencher a lacuna deixada pelas abordagens tradicionais e fornecer uma ferramenta de estratificação de risco mais sensível e individualizada para identificar EP em pacientes com FA com suspeita clínica (Figura Central).

## Métodos

### Desenho do estudo e população

Este estudo retrospectivo foi conduzido no Hospital Oncológico Jinhua Guangfu, um importante centro terciário de saúde em Zhejiang, China. Embora a oncologia seja uma especialidade primária, o hospital funciona como um centro médico abrangente, englobando uma ampla gama de departamentos clínicos, incluindo cardiologia. Os participantes elegíveis foram identificados em todo o hospital por meio de uma consulta sistemática ao sistema de Prontuário Eletrônico do Paciente (PEP) utilizando o código da Classificação Internacional de Doenças, 10ª Revisão (CID-10) para FA (I48). O processo de triagem e seleção está ilustrado na Figura 1. Um total de 545 pacientes consecutivos com FA foram inicialmente identificados no banco de dados do hospital, com o tamanho da amostra determinado pela disponibilidade de pacientes elegíveis durante o período do estudo. Após a exclusão de 274 pacientes com exposição recente à terapia antitrombótica (incluindo anticoagulantes ou antiplaquetários), neoplasia maligna ativa, dados de TEG ausentes ou outros critérios de exclusão, 271 pacientes foram incluídos na análise final. Pacientes que receberam terapia antitrombótica recentemente foram excluídos, pois esses medicamentos podem alterar substancialmente os parâmetros de coagulação derivados da TEG e os parâmetros convencionais, confundindo, assim, a avaliação do perfil de coagulação intrínseco associado ao risco de EP. Embora a terapia antitrombótica de longo prazo seja recomendada para a maioria dos pacientes com FA, na prática clínica hospitalar, uma parcela dos pacientes pode não estar recebendo esse tratamento devido ao diagnóstico recente de FA, à suspensão temporária antes de procedimentos invasivos, ao risco de sangramento ou a outras considerações clínicas. Esses participantes, todos hospitalizados com diagnóstico de FA entre janeiro de 2023 e fevereiro de 2025, incluíam 41 pacientes com EP confirmada e 230 pacientes com FA sem EP. A angioTC foi utilizada como padrão de referência diagnóstico uniforme para EP, a fim de minimizar a heterogeneidade na identificação dos casos; pacientes diagnosticados apenas por outras modalidades não foram incluídos, pois esses métodos são pouco utilizados em nossa instituição e não possuem documentação padronizada nos prontuários médicos. O estudo foi conduzido de acordo com a Declaração de Helsinque, aprovado pelo Comitê de Ética do Hospital Oncológico Jinhua Guangfu (Aprovação nº: 2024-015-01) e relatado em conformidade com a declaração STROBE para estudos observacionais.^17^

**Figura 1:**
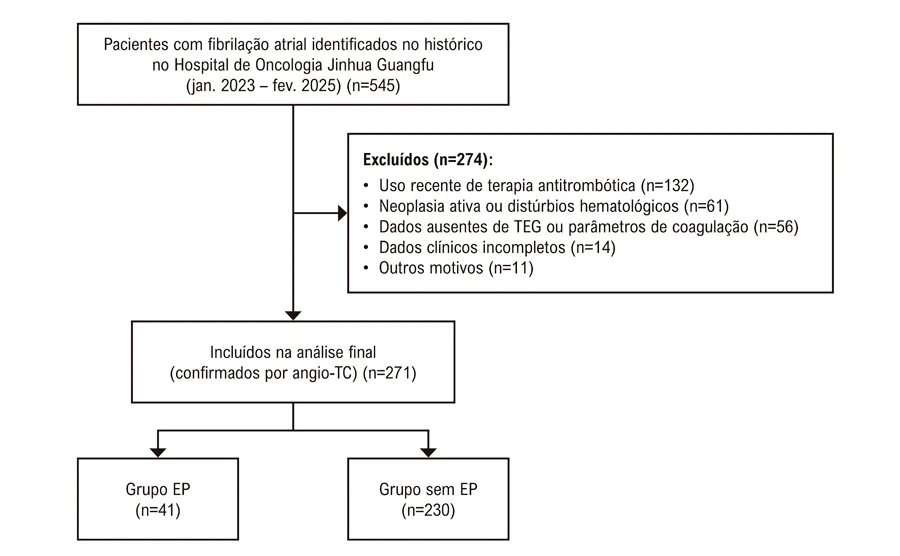
– Fluxograma da seleção dos participantes do estudo.

- **Critérios de inclusão:** (1) Idade ≥ 18 anos; (2) Diagnóstico de FA confirmado de acordo com critérios estabelecidos^18^(3) Submetido a TEG durante a hospitalização e com medições concomitantes de parâmetros de coagulação convencionais; (4) Estado de EP definitivamente determinado por angioTC;^9^ (5) Dados clínicos completos disponíveis.
- **Critérios de exclusão:** (1) Presença de condições subjacentes importantes que afetem a coagulação, como malignidade ativa, trauma agudo, gravidez ou histórico de cirurgia de grande porte; (2) Distúrbios hematológicos diagnosticados, disfunção hepática ou renal grave ou doenças autoimunes ativas; (3) EP claramente secundária a procedimentos cirúrgicos, imobilização prolongada, trauma grave ou terapia de reposição hormonal; (4) Exposição recente à terapia antitrombótica, incluindo agentes anticoagulantes ou antiplaquetários.

### Coleta de dados

Os dados dos pacientes foram obtidos do sistema eletrônico de prontuários médicos do hospital utilizando um formulário padronizado de coleta de dados. As seguintes variáveis foram coletadas para cada paciente incluído no estudo:

**1. Informações demográficas e sobre comorbidades clínicas:**

Sexo, idade, índice de massa corporal (IMC), tempo de internação hospitalar (em dias) e comorbidades, incluindo hipertensão, diabetes mellitus, doença arterial coronariana, trombocitopenia, doença pulmonar obstrutiva crônica (DPOC), insuficiência cardíaca e histórico de infarto cerebral. O escore CHA_2_DS_2_-VASc foi calculado com base nesses parâmetros clínicos.

**2. Parâmetros ecocardiográficos:**

Diâmetro da raiz da aorta (mm), diâmetro do átrio esquerdo (DAE, mm), espessura do septo interventricular (SIV, mm), espessura da parede posterior do ventrículo esquerdo (EPPVE, mm), diâmetro diastólico final do ventrículo esquerdo (DDVE, mm), diâmetro sistólico final do ventrículo esquerdo (DSVE, mm) e fração de ejeção do ventrículo esquerdo (FEVE).

**3. Avaliações laboratoriais:**

Contagem de plaquetas (PLT), índices de função renal, incluindo creatinina sérica (SCr), taxa de filtração glomerular estimada (TFGe) e ácido úrico (AU), perfil lipídico composto por colesterol total (CT), triglicerídeos (TG), colesterol de lipoproteína de alta densidade (HDL-C) e colesterol de lipoproteína de baixa densidade (LDL-C), e proteína C-reativa de alta sensibilidade (PCR-as).

**4. Parâmetros de coagulação convencionais:**

Tempo de protrombina (TP, s), atividade de protrombina (TP%, %), razão normalizada internacional (RNI), tempo de tromboplastina parcial ativada (TTPA, s), tempo de trombina (TT, s), concentração de fibrinogênio (FIB, g/L) e nível de dímero-D (mg/L).

**5. Parâmetros TEG:**

Amostras de sangue venoso periférico foram coletadas de todos os participantes na manhã seguinte à admissão, após jejum noturno. As amostras foram coletadas em tubos a vácuo contendo citrato de sódio a 3,2% como anticoagulante, na proporção de 9:1 (sangue: anticoagulante), e homogeneizadas. Para garantir a confiabilidade dos resultados, todas as amostras foram processadas em até 2 horas após a coleta.

A análise TEG foi realizada utilizando o analisador de TEG UD-T5000, seguindo as instruções do fabricante. Em nossa instituição, embora a TEG não seja realizada rotineiramente em todos os pacientes com FA, ela é comumente utilizada em pacientes hospitalizados com suspeita de condições trombóticas, anormalidades de coagulação inexplicáveis ou quando os médicos necessitam de uma avaliação mais abrangente do estado de coagulação para orientar a tomada de decisões clínicas. Após a ativação, o instrumento registrou continuamente a curva TEG em tempo real e derivou automaticamente parâmetros-chave com base nas alterações das propriedades viscoelásticas do sangue. Esses parâmetros incluíram tempo de reação (R), tempo de coagulação (K), ângulo alfa (ângulo α), amplitude máxima (AM) e porcentagem de lise do coágulo em 30 minutos (LY30). As definições dos parâmetros TEG foram as seguintes:^19,20^

1. **Tempo R:** Duração desde o início do teste até a formação inicial de fibrina, indicada por uma amplitude TEG de 2 mm.
2. **Tempo K:** Intervalo de tempo desde o final de R até o momento em que o coágulo atinge uma força correspondente a uma amplitude de 20 mm.
3. **Ângulo α:** O ângulo formado entre a tangente do segmento ascendente da curva TEG e a linha de base, indicando a taxa de fortalecimento do coágulo.
4. **AM:** A AM da curva TEG, representando a força e a estabilidade máximas do coágulo.
5. **LY30:** Redução percentual na amplitude do coágulo medida 30 minutos após a AM, refletindo a atividade fibrinolítica.

Dois investigadores independentes extraíram dados dos registros médicos eletrônicos. Inconsistências ocorreram em menos de 5% dos casos, principalmente relacionadas à classificação dos padrões de FA devido a notas clínicas ambíguas e à verificação do histórico de medicação pré-internação. Essas inconsistências foram resolvidas por um terceiro revisor por meio de uma análise detalhada do prontuário médico completo do paciente. Todas as informações identificáveis do paciente foram removidas para anonimizar os dados antes da análise.

### Análise de tamanho da amostra e poder estatístico post-hoc

Devido à natureza retrospectiva deste estudo de modelo preditivo, não foi realizado nenhum cálculo formal prospectivo do tamanho da amostra. O rigor metodológico foi mantido pela adesão ao mínimo de 10 eventos por preditor (EPV) para estimativa estável do modelo, complementado por análise de poder post-hoc para confirmar a adequação do tamanho da amostra. O modelo final identificou três preditores independentes para EP em pacientes com FA, com 41 eventos de EP entre 271 indivíduos incluídos, resultando em um EPV de 13,7 — excedendo o limiar recomendado de 10 e corroborando a estimativa confiável dos parâmetros. A análise de poder post-hoc, realizada utilizando o pacote pROC no R, baseou-se na AUC observada de 0,878 e em um tamanho de amostra de 271. Com um nível α bilateral de 0,05, o poder estatístico excedeu 90% para detectar uma AUC significativamente maior que 0,70 (hipótese nula), indicando um tamanho de amostra suficiente para identificar um desempenho preditivo significativo do modelo. Além disso, a validação interna usando 1000 reamostragens boot resultou em um intervalo de confiança estreito para a AUC corrigida para otimismo (0,804–0,916), reforçando a robustez do modelo e a adequação do tamanho da amostra.

### Análise estatística

Todas as análises estatísticas foram realizadas utilizando o software R (versão 4.3.0) e o SPSS (versão 26.0). A normalidade da distribuição dos dados foi avaliada pelo teste de Kolmogorov-Smirnov. Estatísticas descritivas foram utilizadas para resumir os dados. Variáveis contínuas com distribuição normal foram apresentadas como média ± desvio padrão, enquanto dados com distribuição não normal foram expressos como mediana com intervalo interquartil (IIQ). Variáveis categóricas foram descritas como frequências e porcentagens. Comparações entre grupos foram realizadas utilizando o teste t de Student para amostras independentes ou o teste U de Mann-Whitney, conforme apropriado. O teste do qui-quadrado foi utilizado para variáveis categóricas. Utilizou-se a análise de regressão logística multivariada com o método *stepwise* reverso para identificar preditores independentes de EP, e, posteriormente, construiu-se um modelo de nomograma com base no modelo de regressão final. A capacidade discriminatória do modelo foi avaliada utilizando a curva ROC e a AUC, onde uma AUC mais alta indica melhor discriminação. O teste de DeLong foi utilizado para comparar as AUCs entre diferentes modelos ou subgrupos para avaliar a significância estatística das diferenças de discriminação. A calibração do modelo foi avaliada pelo teste de bondade de ajuste de Hosmer-Lemeshow e por curvas de calibração, examinando a concordância entre os resultados previstos e observados. A utilidade clínica foi avaliada usando a ADC, que estima o benefício clínico líquido em uma gama de probabilidades limiares. Para avaliar a robustez e a validade interna do modelo, foi realizada uma reamostragem *bootstrap* (1.000 iterações). A AUC média dos conjuntos de validação foi calculada e uma curva ROC foi gerada para avaliar ainda mais a generalização do modelo. Além disso, uma análise de sensibilidade foi conduzida para avaliar a robustez dos resultados, incorporando variáveis clinicamente relevantes identificadas na análise univariada ao modelo de regressão logística multivariada. Um valor de p bicaudal < 0,05 foi considerado estatisticamente significativo.

## Resultados

### Características basais da população do estudo

Um total de 271 pacientes hospitalizados com FA foram incluídos no estudo, dos quais 41 (15,13%) apresentavam EP concomitante (Tabela 1). As características demográficas basais, incluindo idade, sexo, IMC e tempo de internação hospitalar, foram comparáveis entre os dois grupos. Da mesma forma, o tipo de FA, as comorbidades e o risco tromboembólico (escore CHA_2_DS_2_-VASc mediano: 4,00) não diferiram significativamente. A avaliação ecocardiográfica mostrou que os pacientes com EP apresentavam FEVE significativamente menor em comparação com aqueles sem EP (60,20%)±8,77% contra 63,91±8,81%, p = 0,013), enquanto outros parâmetros ecocardiográficos foram comparáveis entre os grupos. Notavelmente, os níveis de PCR-as foram marcadamente mais elevados em pacientes com EP (mediana de 14,40 vs. 2,92 mg/L, p < 0,001), enquanto outros índices laboratoriais, incluindo função renal, perfil lipídico e contagem de plaquetas, foram semelhantes entre os grupos.

**Tabela 1:** – Comparação das características basais entre o grupo com embolia pulmonar e o grupo sem embolia pulmonar

Variáveis	Total (n = 271)	Não-EP (n = 230)	EP (n = 41)	p
**Idade (anos)**	76,48 ± 10,20	76,00 ± 10,21	77,51±8,64	0.320
**Sexo, n (%)**				0,282
Masc.	153 (56,46)	133 (57,83)	20 (48,78)	
Fem.	118 (43,54)	97 (42,17)	21 (51,22)	
**IMC(kg/m^2^)**	22,86 (20,28, 25,28)	23,00 (20,28, 25,28)	22,39 (20,41, 25,23)	0,618
**Tempo de internação hospitalar (dias)**	7,81 (5,14, 12,92)	7.11 (5.12, 12.92)	8,79 (5,21, 12,16)	0,579
**Padrão FA, n (%)**				0,351
FA paroxística	70 (25,83)	57 (24,78)	13 (31,71)	
FA não paroxística	201 (74,17)	173 (75,22)	28 (68,29)	
**Histórico médico**				
**Hipertensão, n (%)**				0,634
Não	88 (32,47)	76 (33,04)	12 (29.27)	
Sim	183 (67,53)	154 (66,96)	29 (70,73)	
**Diabetes mellitus, n (%)**				0,233
Não	211 (77,86)	182 (79,13)	29 (70,73)	
Sim	60 (22.14)	48 (20,87)	12 (29.27)	
**Doença arterial coronariana, n (%)**				0,386
Não	122 (45,02)	101 (43,91)	21 (51,22)	
Sim	149 (54,98)	129 (56,09)	20 (48,78)	
**Trombocitopenia, n (%)**				0,819
Não	228 (84,13)	194 (84,35)	34 (82,93)	
Sim	43 (15,87)	36 (15,65)	7 (17.07)	
**DPOC, n (%)**				0,907
Não	223 (82,29)	189 (82,17)	34 (82,93)	
Sim	48 (17,71)	41 (17,83)	7 (17.07)	
**Insuficiência cardíaca, n (%)**				0,782
Não	235 (86,72)	200 (86,96)	35 (85,37)	
Sim	36 (13,28)	30 (13.04)	6 (14,63)	
**Infarto cerebral, n (%)**				0,296
Não	179 (66,05)	149 (64,78)	30 (73,17)	
Sim	92 (33,95)	81 (35,22)	11 (26,83)	
**Tumor maligno, n (%)**				0,587
Não	226 (83,39)	193 (83,91)	33 (80,49)	
Sim	45 (16,61)	37 (16.09)	8 (19,51)	
**Escore CHA_**2**_DS_**2**_-VASc**	4,00 (3,00, 6,00)	4,00 (3,00, 6,00)	4,00 (3,00, 5,00)	0,474
**Parâmetros de laboratório**				
PLT (×109/L)	166,00 (128,50, 212,00)	168,50 (129,00, 215,75)	157,00 (125,00, 196,00)	0,338
PCR-as (mg/L)	3,80 (1,19, 12,60)	2,92 (0,97, 9,96)	14,40 (3,50, 35,76)	<0,001
AU (μmol/L)	310,00 (236,00, 379,00)	309,00 (238,00, 369,00)	317,00 (189,00, 441,00)	0,642
SCr (μmol/L)	78,00 (64,00, 103,00)	79,50 (65,00, 99,25)	74,00 (61,00, 124,00)	0,923
TFGe (mL/min/1,73m^2^)	77,26 (57,97, 97,03)	76,98 (58,83, 96,21)	79,67 (45,00, 109,28)	0,818
TG (mmol/L)	1,12 (0,80, 1,60)	1,11 (0,79, 1,60)	1,17 (0,83, 1,60)	0,600
CT (mmol/L)	3,73 (3,11, 4,52)	3,69 (3,11, 4,56)	3,82 (3,09, 4,39)	0,787
HDL-C (mmol/L)	1,18 (0,97, 1,39)	1,20 (0,98, 1,40)	1,07 (0,85, 1,28)	0,099
LDL-C (mmol/L)	2,16 (1,70, 2,80)	2,15 (1,68, 2,76)	2,25 (1,87, 2,96)	0,319
**Parâmetros ecocardiográficos**				
Diâmetro da raiz da aorta	27,18 ± 4,09	27,06 ± 4,25	27,94 ± 2,87	0,152
DAE (mm)	39,71 ± 8,04	39,57 ± 7,92	40,55 ± 8,83	0,530
SIV (mm)	8,94 ± 1,38	8,99 ± 1,41	8,65 ± 1,14	0,191
EPVE (mm)	8,96 ± 1,34	9,02 ± 1,32	8,65 ± 1,38	0,153
DDVE (mm)	47,50 ± 6,72	47,64 ± 6,62	46,71 ± 7,38	0,478
DSVE (mm)	30,54 ± 6,50	30,63 ± 6,55	30,00 ± 6,26	0,620
FEVE (%)	63,35 ± 8,89	63,91 ± 8,81	60,20 ± 8,77	0,013

A trombocitopenia foi definida como uma contagem de plaquetas < 100 × 10⁹/L. EP: embolia pulmonar; IMC: índice de massa corporal; DPOC: doença pulmonar obstrutiva crônica; DAE: diâmetro do átrio esquerdo; SIV: espessura do septo interventricular; EPVE: espessura da parede posterior do ventrículo esquerdo; DDVE: diâmetro diastólico final do ventrículo esquerdo; DSVE: diâmetro sistólico final do ventrículo esquerdo.

### Comparação dos parâmetros TEG e marcadores de coagulação entre os grupos

Conforme demonstrado na Tabela 2, foram encontradas diferenças significativas nos parâmetros do TEG e nos marcadores de coagulação entre os dois grupos. Os pacientes do grupo com EP apresentaram ângulo α e AM significativamente maiores em comparação com os do grupo sem EP. Além disso, o tempo R, o tempo K e o LY30 apresentaram diferenças significativas entre os grupos. Os níveis de FIB e dímero-D também estavam acentuadamente elevados no grupo com EP. Em contrapartida, não foram observadas diferenças significativas no TP, na atividade da TP, no RNI, no TTP) ou no TT entre os grupos.

**Tabela 2:** – Comparação dos parâmetros TEG e indicadores de coagulação entre os grupos de EP e sem EP

Variáveis	Total (n = 271)	Não-EP (n = 230)	EP (n = 41)	p
TP(s)	15,12 ± 3,93	15,16 ± 3,97	14,88 ± 3,74	0,679
Atividade TP (%)	70,83 ± 19,24	70,71 ± 19,56	71,48 ± 17,59	0,815
RNI	1,32 ± 0,40	1,32 ± 0,40	1,30 ± 0,40	0,759
TTPA(s)	30,76 ± 6,37	30,96 ± 6,39	29,65 ± 6,22	0,228
TT(s)	16,06 ± 1,31	16,06 ± 1,24	16,05 ± 1,68	0,986
FIB (g/L)	3,39 (2,43, 4,75)	3,26 (2,39, 4,52)	4,77 (2,68, 6,62)	0,001
D-dímero (mg/L)	0,82 (0,54, 1,20)	0,74 (0,48, 1,06)	1,12 (0,79, 2,93)	<0,001
Tempo R (min)	3,70 (2,90, 4,60)	3,80 (3,00, 4,70)	3,20 (2,60, 3,80)	0,001
Tempo K (min)	1,20 (0,90, 1,60)	1,30 (1,00, 1,70)	1,10 (0,80, 1,30)	0,002
LY30 (%)	1,10 (0,10, 3,20)	1,20 (0,10, 3,60)	0,80 (0,00, 1,60)	0,026
Ângulo α (°)	69,54 ± 5,72	69,14 ± 5,94	71,81 ± 3,57	<0,001
AM (mm)	56,73 ± 4,57	56,00 ± 4,00	60,81 ± 5,40	<0,001

TEG: tromboelastografia; PT: tempo de protrombina; RNI: razão normalizada internacional; TTPA: tempo de tromboplastina parcial ativada; TT: tempo de trombina; FIB: fibrinogênio; AM: amplitude máxima; LY30: lise em 30 minutos; ângulo α: velocidade de fortalecimento do coágulo; R: tempo de reação; K: cinética do coágulo.

### Fatores de risco independentes para EP em pacientes com FA

A análise de regressão logística univariada identificou o tempo R, o tempo K, o ângulo α, LY30, AM, FIB e dímero-D como fatores significativos associados à EP em pacientes com FA. A análise multivariada subsequente, utilizando regressão *stepwise backward*, revelou que AM, FIB e dímero-D foram fatores de risco independentes para EP em pacientes com FA. As demais variáveis não mantiveram significância estatística no modelo multivariado (Tabela 3).

**Tabela 3:** – Análise de regressão logística univariada e multivariada dos fatores de risco para EP em pacientes com FA

Variáveis	Análise Univariada		Análise Multivariada	
	p	OR (IC 95%)	p	OR (IC 95%)
Tempo R (min)	0,002	0,62 (0,46 ~ 0,84)		
Tempo K (min)	0,010	0,32 (0,13 ~ 0,76)		
Ângulo α (°)	0,007	1,10 (1,03 ~ 1,18)		
AM (mm)	<0,001	1,30 (1,18 ~ 1,43)	<0,001	1,37 (1,21 ~ 1,54)
LY30(%)	0,005	0,72 (0,57 ~ 0,91)		
FIB (g/L)	<0,001	2,20 (1,57 ~ 3,09)	<0,001	2,30 (1,46 ~ 3,62)
D-dímero (mg/L)	<0,001	2,55 (1,78 ~ 3,65)	<0,001	2,81 (1,77 ~ 4,47)

OR: odds ratio; IC: intervalo de confiança; AM: amplitude máxima; FIB: fibrinogênio; LY30: lise em 30 minutos.

### Construção de nomograma para predição de embolia pulmonar em pacientes com fibrilação atrial

Com base nos resultados da análise de regressão logística multivariada, foi construído um nomograma para prever o risco de EP em pacientes com FA (Figura 2). O modelo incorporou três preditores independentes: dímero-D, FIB e TEG-AM. Para utilizar o nomograma clinicamente, o valor de cada preditor é inicialmente localizado em seu respectivo eixo de variáveis. Uma linha vertical é traçada para cima até o eixo “Pontos” para determinar a pontuação específica para essa variável. A soma dessas pontuações individuais constitui a “Pontuação Total”, que é então localizada no eixo “Pontuação Total”. Finalmente, uma linha vertical é traçada para baixo a partir do eixo “Pontuação Total” até o eixo “Risco” para obter a probabilidade predita individual.

**Figura 2:**
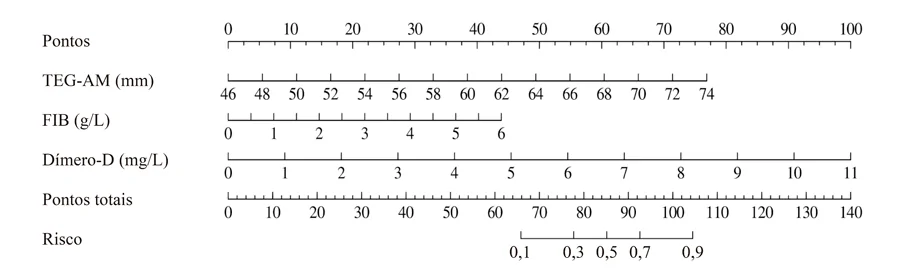
– Nomograma para prever a probabilidade de embolia pulmonar em pacientes com fibrilação atrial.

### Desempenho e validação do modelo

Para avaliar a capacidade discriminativa do nomograma, foi traçada uma curva ROC. A AUC foi de 0,878 (IC 95%: 0,806–0,949), indicando excelente desempenho preditivo (Figura 3A). No valor de corte ideal de 0,204, o modelo apresentou sensibilidade de 87,8% e especificidade de 78,0%. Comparado aos preditores individuais (AM, FIB e dímero-D), o nomograma demonstrou uma AUC significativamente maior (Tabela 4), sugerindo que a combinação de múltiplos fatores pode aumentar substancialmente a precisão preditiva.

**Figura 3:**
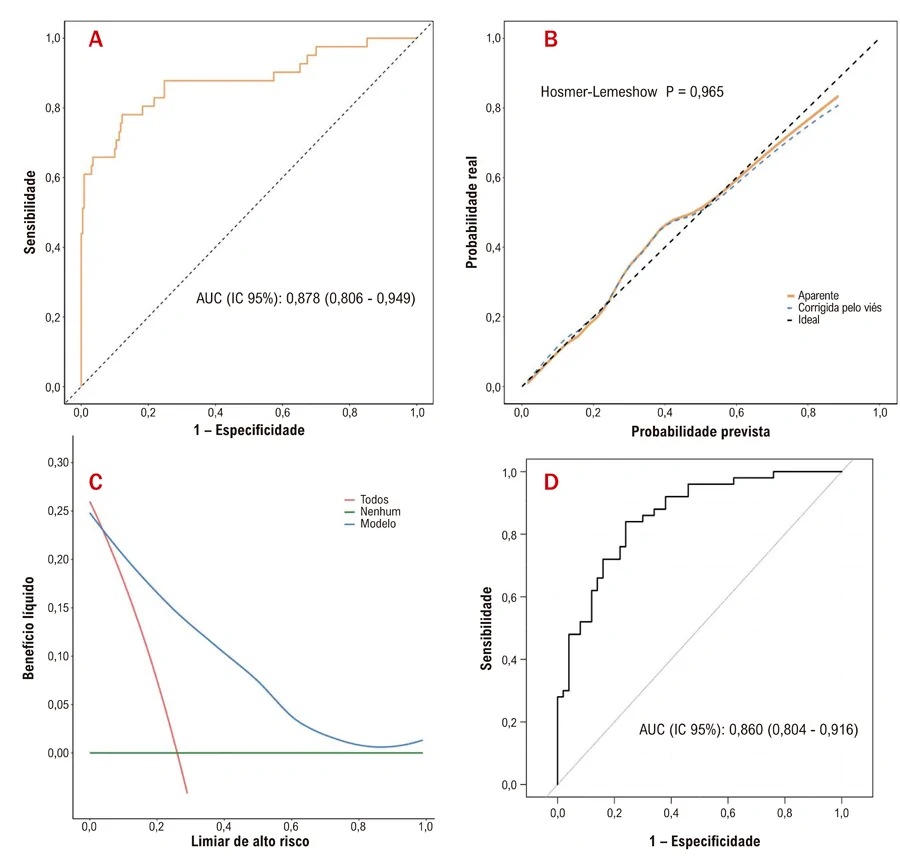
– Avaliação do desempenho do modelo de nomograma para EP em pacientes com FA. (A) Curva ROC do modelo de nomograma; (B) Curva de calibração do modelo de nomograma; (C) Curva ADC; (D) Curva ROC após validação interna usando o método Bootstrap.

**Tabela 4:** – Comparação do desempenho preditivo entre o modelo de nomograma e biomarcadores individuais

Variáveis	AUC (IC 95%)	Sensibilidade	Especificidade	Corte	Teste de Delong
Nomograma	0,878 (0,806-0,949)	87.8%	78,0%	0,204	referência
TEG-AM (mm)	0,771 (0,680-0,862)	72.6%	73.2%	58,2	<0,001
FIB (g/L)	0,741 (0,669-0,813)	70.0%	70.7%	3.199	<0,001
Dímero-D (mg/L)	0,742 (0,662-0,821)	95,2%	41,5%	2.011	<0,001

AUC: área sob a curva; IC: intervalo de confiança; AM: amplitude máxima; FIB: fibrinogênio.

Em termos de calibração, o gráfico de calibração (Figura 3B) demonstrou boa concordância entre as probabilidades previstas e os resultados observados. O teste de bondade de ajuste de Hosmer-Lemeshow apresentou um valor de P de 0,965 (>0,05), corroborando ainda mais o bom ajuste do modelo na coorte de treinamento.

Para avaliar a utilidade clínica do nomograma, foi realizada uma ADC). A curva da ADC (Figura 3C) mostrou que, dentro de uma faixa de probabilidade limite de 0,2 a 0,8, o modelo proporcionou um benefício líquido maior do que as estratégias de “tratar todos” ou “não tratar ninguém”. O benefício líquido máximo foi observado em um limiar de 0,3, sugerindo que o modelo poderia ajudar a evitar aproximadamente 120 intervenções desnecessárias por 1.000 pacientes — demonstrando forte aplicabilidade clínica.

Além disso, para validar a robustez do modelo, foi realizada uma validação interna utilizando *bootstrapping* com 1.000 reamostragens. A AUC média obtida foi de 0,860 (IC 95%: 0,804–0,916), indicando boa generalização e estabilidade do modelo (Figura 3D).

### Análise de sensibilidade e comparação de modelos

Para avaliar ainda mais a robustez de nossos achados e o potencial valor agregado das características clínicas, realizamos uma análise de sensibilidade. Nessa análise, variáveis clínicas com p < 0,05 na análise univariada (especificamente, PCR-as e FEVE) foram introduzidas no modelo de regressão logística multivariada juntamente com os parâmetros de coagulação. Nesse modelo ampliado, a FEVE foi identificada como um preditor independente significativo (OR: 0,95, p = 0,027), enquanto a PCR-as não manteve a significância. O modelo ampliado (combinando AM, fibrinogênio, dímero-D e FEVE) apresentou uma AUC de 0,893 (IC 95%: 0,830-0,957). No entanto, o teste de DeLong demonstrou que essa melhora não foi estatisticamente significativa em comparação com o nosso modelo original de três variáveis (AUC 0,878 vs. 0,893, p = 0,057). Portanto, considerando o princípio da parcimônia do modelo e da utilidade clínica – em que o modelo original requer apenas uma única coleta de sangue, sem a necessidade de ecocardiografia imediata – mantivemos o modelo baseado na coagulação (AM, fibrinogênio, dímero-D) para a construção do nomograma final.

## Discussão

Neste estudo, desenvolvemos um modelo de nomograma para prever o risco de EP em pacientes hospitalizados com FA, integrando parâmetros de TEG com índices de coagulação convencionais. Os resultados demonstraram que os níveis de AM, FIB e dímero-D foram fatores de risco independentes para EP, e o modelo construído apresentou bom desempenho preditivo. Devido à sua natureza não invasiva, facilidade de uso e adequação para aplicação à beira do leito, este modelo fornece uma nova ferramenta para a identificação precoce de EP de alto risco em pacientes com FA.

A prevalência de EP em nosso estudo foi de 15,1%, o que é notavelmente maior do que a relatada em populações gerais com FA. Por exemplo, um estudo multicêntrico na China realizado por Bai et al.^21^ relataram uma prevalência de EP intra-hospitalar de 1,2% entre todos os pacientes com FA. Da mesma forma, estudos populacionais de grande escala realizados por Friberg et al.^22^e Hald et al.^6^observaram taxas de incidência muito mais baixas, variando de 2,9 a 18,5 por 1.000 pessoas-ano. Essa discrepância em nosso estudo deve-se principalmente aos critérios de inclusão específicos utilizados: restringimos nossa análise a pacientes submetidos à angioTC, um exame diagnóstico normalmente reservado para indivíduos com alta suspeita clínica de EP (por exemplo, dispneia inexplicada, hipoxemia ou dímero-D elevado). Consequentemente, nossa população de estudo representa uma coorte de “alto risco com suspeita de EP”, e não uma população geral de rastreamento de FA. Essa distinção é crucial, pois nosso modelo foi especificamente projetado para auxiliar os médicos a confirmar ou descartar EP nesse grupo desafiador de pacientes com suspeita da doença.

Níveis elevados de dímero-D foram identificados como um preditor independente de EP em pacientes com FA, corroborando estudos anteriores.^23,24^Como produto da degradação da fibrina, o dímero-D indica coagulação e fibrinólise ativas.^25^Embora a especificidade do dímero-D diminua com a idade, nosso estudo demonstra que a integração deste com TEG-AM e fibrinogênio aumenta significativamente a precisão diagnóstica em comparação com o dímero-D isoladamente. Essa abordagem multiparamétrica mitiga efetivamente a taxa de falsos positivos frequentemente observada em populações idosas. Do ponto de vista fisiopatológico, a ligação entre FA e EP é principalmente indireta.^6^Embora a embolia paradoxal por meio de um *shunt* intracardíaco, como um forame oval patente, represente uma via direta, a FA mais comumente promove um estado sistêmico de hipercoagulabilidade e pró-inflamatório, prejudica o débito cardíaco e causa estase venosa. Esses fatores aumentam o risco de trombose venosa profunda e trombose venosa profunda in situ do coração direito, que, por sua vez, servem como as principais fontes de EP.^26-28^Grandes estudos de coorte confirmam que a FA eleva significativamente o risco de EP e acidente vascular cerebral isquêmico, particularmente nos primeiros 6 meses após o diagnóstico.^6^Além disso, a FA está associada à hipertensão pulmonar, que pode agravar a função do ventrículo direito e promover a trombogênese, criando um ciclo vicioso.^26^Assim, os níveis elevados de dímero-D, fibrinogênio e AM em nosso modelo provavelmente refletem essa tendência trombótica sistêmica. É importante ressaltar que, como nosso estudo excluiu pacientes em uso de anticoagulantes, esses parâmetros fornecem uma avaliação direta do estado de hipercoagulabilidade intrínseca em pacientes com FA, identificando aqueles com maior risco de EP, principalmente pela via do TEV.

O FIB é um substrato fundamental na cascata de coagulação, e seus níveis elevados indicam um estado de hipercoagulabilidade, contribuindo tanto para a formação quanto para a estabilização do trombo.^29^Estudos demonstraram uma associação significativa entre níveis elevados de FIB e o risco de TEV.^30,31^A própria FA representa uma condição inflamatória crônica de baixo grau, e evidências anteriores demonstraram níveis persistentemente elevados de marcadores inflamatórios, como proteína C-reativa e interleucina-6. Esses mediadores inflamatórios podem induzir a síntese hepática de FIB e fatores de coagulação, aumentando assim o ambiente protrombótico.^32,33^Além disso, níveis elevados de FIB podem aumentar a viscosidade sanguínea e promover a agregação de eritrócitos, o que agrava o fluxo sanguíneo lento e cria um ambiente favorável à trombogênese.^34^Esses mecanismos, em conjunto, predispõem pacientes com FA à EP em um contexto de níveis elevados de fibrinogênio.

Além disso, observamos que pacientes com EP apresentaram níveis de proteína C-reativa de alta sensibilidade (PCR-as) significativamente mais elevados do que aqueles sem EP. Esse achado reforça o papel potencial da inflamação sistêmica no estado protrombótico de pacientes com FA e EP. A PCR-as elevada pode refletir uma atividade inflamatória exacerbada associada ao aumento da expressão de fibrinogênio na FA, bem como processos tromboinflamatórios relacionados ao endotélio e às plaquetas que podem contribuir para a formação de trombos. Embora a PCR-as não tenha sido mantida como um preditor independente no modelo multivariado após o ajuste para parâmetros de coagulação, sua elevação acentuada no grupo com EP ressalta a estreita interação entre inflamação e coagulação nessa população de alto risco.^35,36^

A AM é um parâmetro crítico derivado da TEG que reflete a força geral do coágulo, determinada tanto pela função plaquetária quanto pela estrutura da fibrina.^37,38^Em nosso estudo, a elevação da AM foi significativamente associada à EP relacionada à FA, sugerindo um papel fundamental da ativação plaquetária na trombogênese nesses pacientes. Notavelmente, embora a trombocitopenia possa teoricamente reduzir a AM, sua prevalência foi equilibrada entre os grupos em nosso estudo (P=0,819). Isso indica que a elevação observada da AM no grupo com EP reflete um estado de hipercoagulabilidade genuíno — provavelmente impulsionado pelo aumento da função plaquetária e do fibrinogênio — em vez de um viés introduzido pela contagem de plaquetas. Assim, a AM fornece um marcador funcional do potencial trombótico, complementando as informações estáticas fornecidas pela contagem de plaquetas. Pesquisas anteriores demonstraram que a AM pode predizer complicações trombóticas após cirurgia ortopédica e em pacientes com sangramento gastrointestinal.^39,40^Na FA, as alterações hemodinâmicas nos átrios frequentemente resultam em estase sanguínea local e turbulência, o que promove a formação de fibrina e a agregação plaquetária, levando a trombos mais densos e estáveis.^29,41^Uma vez desprendidos, esses trombos podem viajar pela circulação sanguínea e obstruir as artérias pulmonares, resultando em EP.^9,42^Por ser um indicador sensível da viscoelasticidade sanguínea, a AM pode servir como um valioso complemento para a estratificação precoce do risco de EP na população com FA.

Além disso, contextualizamos nosso nomograma baseado em TEG em relação às ferramentas preditivas existentes. Os escores clínicos tradicionais (por exemplo, Wells, Genebra) apresentam especificidade limitada em pacientes idosos com FA devido à sobreposição de sintomas. Modelos recentes exploraram abordagens diferentes: um nomograma clínico de Ling et al.^7^ alcançou uma AUC de 0,721, enquanto um modelo de IA-ECG de Silva et al.^43^ apresentou alta especificidade (100%), mas baixa sensibilidade (50%), com uma AUC de 0,75. Estudos epidemiológicos associaram a gravidade da FA (via escore CHA_2_DS_2_-VASc) ao risco de TEV,^32,44^no entanto, esse escore em si demonstrou utilidade preditiva modesta para EP (AUC 0,62). Em contraste, nosso modelo integra o marcador funcional dinâmico TEG-AM, resultando em discriminação superior (AUC 0,878) com sensibilidade equilibrada de 87,8% e especificidade de 78,0%. Isso sugere que a avaliação da função de coagulação em tempo real oferece maior valor preditivo nessa coorte de alto risco.

A relação custo-benefício da incorporação da TEG no manejo da FA requer consideração. Embora a TEG seja mais cara do que os testes de coagulação de rotina (p. ex., TP, TTPA), permanece substancialmente mais barata do que a angiotomografia computadorizada pulmonar (angioTC). A aplicação indiscriminada a todos os pacientes ambulatoriais com FA pode não ser economicamente viável. No entanto, nosso modelo se concentra em um subgrupo definido de pacientes internados de alto risco com suspeita clínica de EP, cuja prevalência da doença em nosso estudo foi de 15,1%. Nesse contexto específico, o uso do nosso nomograma para estratificação inicial de risco pode ser custo-efetivo. Ao identificar com precisão os pacientes de maior risco, ele pode orientar o uso seletivo da angioTC confirmatória, reduzindo exames desnecessários, minimizando a exposição à radiação/contraste e otimizando a alocação de recursos em comparação com uma estratégia de imagem universal.^45,46^

### Pontos fortes e limitações

Este estudo oferece diversas vantagens clínicas. Primeiro, a TEG proporciona uma avaliação dinâmica, reprodutível e acessível à beira do leito da coagulação, complementando os marcadores de coagulação estáticos tradicionais. Segundo, este é o primeiro estudo, até onde sabemos, a construir um modelo de predição de risco de EP especificamente para a população com FA, baseado em AM, FIB e dímero-D. A abordagem multiparamétrica aumenta a robustez e a precisão do modelo, particularmente em cenários com sintomas atípicos ou acesso limitado a exames de imagem.

No entanto, algumas limitações devem ser reconhecidas. Primeiro, este foi um estudo retrospectivo unicêntrico com uma amostra relativamente pequena. A identificação dos pacientes com base na codificação da CID-10 pode estar sujeita a captura incompleta de casos e classificação incorreta, potencialmente introduzindo viés de seleção. Segundo, é importante notar que o estudo foi conduzido em um hospital oncológico especializado. Mesmo com a exclusão rigorosa de neoplasias malignas ativas, a população de pacientes nesse contexto específico apresenta inerentemente maior fragilidade e uma carga mais elevada de comorbidades complexas em comparação com coortes em hospitais gerais. Consequentemente, esse contexto específico pode limitar a generalização de nossos achados para populações de menor risco. Terceiro, devido à natureza retrospectiva da coleta de dados, diversas variáveis clinicamente importantes relacionadas à EP — incluindo histórico prévio de TEV, status de trombofilia, sinais e sintomas detalhados de EP e achados ecocardiográficos do coração direito (incluindo tamanho e função das câmaras) — não estavam consistentemente disponíveis nos prontuários médicos e, portanto, não puderam ser incluídas na análise. Em quarto lugar, devido ao número limitado de eventos de EP, não realizamos análises de subgrupos para comorbidades específicas, como insuficiência cardíaca ou infarto cerebral prévio. Recomenda-se a realização de estudos futuros com coortes maiores para explorar melhor os perfis de risco específicos desses subgrupos. Em quinto lugar, o modelo carece de validação externa e sua aplicabilidade em diversas populações permanece incerta. Por fim, não havia dados de acompanhamento em longo prazo disponíveis, o que impossibilitou a avaliação do valor preditivo a longo prazo. Pesquisas futuras devem adotar um delineamento prospectivo e multicêntrico com coortes maiores, incorporar biomarcadores adicionais e explorar a utilidade do modelo em condições tromboembólicas mais amplas.

## Conclusão

Em resumo, este estudo desenvolveu com sucesso um modelo nomográfico para prever EP em pacientes com FA, baseado em dímero-D, fibrinogênio e AM. O modelo demonstrou desempenho preditivo satisfatório e aplicabilidade clínica, oferecendo uma ferramenta valiosa para a identificação precoce de pacientes de alto risco e a formulação de estratégias de tratamento individualizadas. Estudos prospectivos multicêntricos de grande escala são necessários para validar e aprimorar ainda mais este modelo preditivo.
