# Supplementary material for: Um Modelo Preditivo para Embolia Pulmonar em Pacientes com Fibrilação Atrial Baseado em Tromboelastografia e Parâmetros de Coagulação
Source: Arq Bras Cardiol. 2026 Aug 24;123(8):e20250683. [Article in Portuguese] doi: 10.36660/abc.20250683 (PMC13718333; doi:10.36660/abc.20250683)
Supplement: Translation

# Translation: A Predictive Model for Pulmonary Embolism in Patients with Atrial Fibrillation Based on Thromboelastography and Coagulation Parameters

**DOI:** 10.36660/abc.20250683i

**Authors:** Xingqi Huang, Jianmei Liu

**Affiliations:** 1 China Department of Blood Transfusion, Jinhua Guangfu Oncology Hospital, Jinhua – China; 2 China Department of Cardiovascular Medicine, Jinhua Guangfu Oncology Hospital, Jinhua – China

**Keywords:** Atrial Fibrillation, Pulmonary Embolism, Thrombelastography

## Abstract

**Background:**

Patients with atrial fibrillation (AF) face an elevated risk of pulmonary embolism (PE), yet existing prediction tools demonstrate limited accuracy.

**Objectives:**

This study aimed to develop and validate a novel predictive model integrating thromboelastography (TEG) parameters with conventional coagulation markers for PE risk assessment in AF patients.

**Methods:**

We conducted a retrospective study of 271 hospitalized AF patients who underwent CTPA for suspected PE (41 with PE, 230 without). The mean age was 76.5 years, 56.5% were male, and hypertension was the most common comorbidity (67.5%). PE diagnosis was confirmed by computed tomography pulmonary angiography. Baseline characteristics, TEG parameters (including reaction time, maximum amplitude [MA], and α-angle), and standard coagulation markers (D-dimer, fibrinogen) were analyzed. Statistical significance was defined as a two-sided P-value < 0.05. Multivariate logistic regression identified independent predictors, which were incorporated into a nomogram. Model performance was evaluated using receiver operating characteristic (ROC) analysis, calibration curves, and decision curve analysis (DCA).

**Results:**

The prevalence of PE was 15.1%. Patients with PE exhibited significantly higher TEG-MA, D-dimer, and fibrinogen levels compared to the non-PE group (all P<0.05). Multivariate analysis identified these three markers as independent predictors. The nomogram demonstrated excellent discrimination (AUC=0.878, 95% CI:0.806-0.949), with 87.8% sensitivity and 78.0% specificity at the optimal cutoff. The model showed good calibration (Hosmer-Lemeshow p=0.965) and significant clinical utility.

**Conclusion:**

The TEG-based nomogram combining MA, D-dimer, and fibrinogen provides accurate, bedside-accessible PE risk stratification for AF patients. This tool may facilitate early identification of high-risk individuals and guide clinical decision-making regarding anticoagulation therapy. Further prospective studies are warranted to validate these findings.

## Introduction

Atrial fibrillation (AF), the most common sustained cardiac arrhythmia in clinical practice, is becoming increasingly prevalent with global population aging and has emerged as a major public health concern. Recent guidelines from the European Society of Cardiology and the American Heart Association/American College of Cardiology emphasize the importance of assessing complications associated with AF.^1,2^ PE is a serious yet often underrecognized complication in AF patients. Studies have shown that the incidence of PE was 1.55 per 1,000 person-years in patients with AF, representing a substantially higher risk compared to those without AF.^3^ The coexistence of PE significantly prolongs hospitalization and increases mortality, especially among elderly patients and those with multiple comorbidities, leading to markedly worse short- and long-term outcomes.^4,5^ Moreover, the nonspecific clinical presentations of both AF and PE—such as dyspnea and palpitations—can easily lead to missed or incorrect diagnoses, thereby exacerbating disease burden and healthcare resource consumption^6^

Currently, diagnosing PE in AF patients remains clinically challenging. Definitive diagnosis of PE primarily relies on imaging, with computed tomography pulmonary angiography (CTPA) serving as the gold standard. While CTPA has high diagnostic value, it also carries limitations such as ionizing radiation exposure, risk of contrast-induced nephropathy, and potential overdiagnosis of subsegmental emboli.^7^ Among laboratory markers, D-dimer is commonly used for auxiliary screening due to its high sensitivity. However, its specificity is low, and it is easily influenced by factors such as age, comorbidities, and inflammatory states, which can lead to increased false-positive rates.^8^ Clinical scoring systems such as the Wells and Geneva scores are useful in the general population, but their performance may be reduced in complex hospitalized patients with overlapping cardiopulmonary symptoms and multiple comorbidities.^9,10^ These limitations underscore the need for more reliable, dynamic, and reproducible biomarkers to aid in the early identification of PE risk among AF patients, ultimately improving clinical decision-making and prognostic management.

TEG is a dynamic assay that evaluates the entire coagulation process, providing comprehensive insights into coagulation, fibrinolysis, and platelet function.^11,12^ Unlike conventional coagulation tests, TEG enables real-time monitoring of a patient’s coagulation status and has been widely used for bleeding and thrombotic risk assessment in trauma, oncology, and critical care settings.^13-15^ In recent years, preliminary studies have explored the utility of TEG in predicting venous thromboembolism (VTE), suggesting its potential for early identification.^16^ However, evidence regarding the application of TEG in AF—a population at particularly high thrombotic risk—remains limited. Therefore, constructing a predictive model based on TEG parameters in combination with conventional laboratory indices may help bridge the gap left by traditional approaches and provide a more sensitive and individualized risk stratification tool for identifying PE among AF patients with clinical suspicion (Central Illustration).

## Methods

### Study design and population

This retrospective study was conducted at Jinhua Guangfu Oncology Hospital, a major tertiary-care facility in Zhejiang, China. Although oncology is a primary specialty, the hospital operates as a comprehensive medical center, encompassing a wide range of clinical departments, including cardiology. Eligible participants were identified hospital-wide through a systematic query of the Electronic Medical Record (EMR) system using the International Classification of Diseases, 10th Revision (ICD-10) code for atrial fibrillation (I48). The screening and selection process is illustrated in Figure 1. A total of 545 consecutive patients with AF were initially identified from the hospital-wide database, with the sample size determined by the availability of eligible patients during the study period. After excluding 274 patients with recent exposure to antithrombotic therapy (including anticoagulant or antiplatelet agents), active malignancy, missing TEG data, or other exclusion criteria, 271 patients were included in the final analysis. Patients receiving recent antithrombotic therapy were excluded because these medications could substantially alter TEG-derived and conventional coagulation parameters, thereby confounding the evaluation of the intrinsic coagulation profile associated with PE risk. Although long-term antithrombotic therapy is recommended for most patients with AF, in real-world inpatient settings, a subset of patients may not be receiving such treatment due to newly recognized AF, temporary withholding before invasive procedures, bleeding risk, or other clinical considerations. These participants, all hospitalized with a diagnosis of AF between January 2023 and February 2025, comprised 41 patients with confirmed PE and 230 patients with AF without PE. CTPA was used as the uniform diagnostic reference standard for PE to minimize heterogeneity in case ascertainment; patients diagnosed only by alternative modalities were not included because these methods were used infrequently in our institution and lacked standardized documentation in the medical records. The study was conducted in accordance with the Declaration of Helsinki, approved by the Ethics Committee of Jinhua Guangfu Oncology Hospital (Approval No.: 2024-015-01), and reported in accordance with the STROBE statement for observational Studies.^17^

**Figure 1:** – Flowchart of study participant selection.

- **Inclusion Criteria:** (1) Age ≥ 18 years; (2) Diagnosis of AF confirmed according to established criteria;^18^ (3) Underwent TEG testing during hospitalization and had concurrent measurements of conventional coagulation parameters; (4) PE status definitively determined by CTPA;^9^ (5) Complete clinical data available.
- **Exclusion Criteria:** (1) Presence of major underlying conditions known to affect coagulation, such as active malignancy, acute trauma, pregnancy, or history of major surgery; (2) Diagnosed hematological disorders, severe hepatic or renal dysfunction, or active autoimmune diseases;(3) PE clearly secondary to surgical procedures, prolonged immobilization, severe trauma, or hormone replacement therapy; (4) Recent exposure to antithrombotic therapy, including anticoagulant or antiplatelet agents.

### Data collection

Patient data were retrieved from the hospital’s electronic medical record system using a standardized data collection form. The following variables were collected for each enrolled patient:

**1. Demographic and clinical comorbidity information:**

Sex, age, body mass index (BMI), length of hospital stay (days), and comorbid conditions, including hypertension, diabetes mellitus, coronary artery disease, thrombocytopenia, chronic obstructive pulmonary disease (COPD), heart failure, and a history of cerebral infarction. The CHA_2_DS_2_-VASc score was calculated based on these clinical parameters.

**2. Echocardiographic Parameters:**

Aortic root diameter (mm), left atrial diameter (LAD, mm), interventricular septal thickness (IVS, mm), left ventricular posterior wall thickness (LVPW, mm), left ventricular end-diastolic diameter (LVEDD, mm), left ventricular end-systolic diameter (LVESD, mm), and left ventricular ejection fraction (LVEF).

**3. Laboratory Assessments:**

Platelet count (PLT), renal function indices including serum creatinine (SCr), estimated glomerular filtration rate (eGFR), and uric acid (UA), lipid profiles consisting of total cholesterol (TC), triglycerides (TG), high-density lipoprotein cholesterol (HDL-C), and low-density lipoprotein cholesterol (LDL-C), and high-sensitivity C-reactive protein (Hs-CRP).

**4. Conventional Coagulation Parameters:**

Prothrombin time (PT, s), prothrombin activity (PT%, %), international normalized ratio (INR), activated partial thromboplastin time (APTT, s), thrombin time (TT, s), fibrinogen concentration (FIB, g/L), and D-dimer level (mg/L).

**5. TEG Parameters:**

Peripheral venous blood was collected from all participants on the morning following admission after an overnight fast. Samples were drawn into vacuum tubes containing 3.2% sodium citrate anticoagulant at a 9:1 blood-to-anticoagulant ratio and mixed thoroughly. To maintain result reliability, all specimens were processed within 2 hours of collection.

TEG analysis was performed using the UD-T5000 thromboelastography analyzer, following the manufacturer’s instructions. At our institution, although TEG is not routinely performed in all patients with atrial fibrillation, it is commonly used in hospitalized patients with suspected thrombotic conditions, unexplained coagulation abnormalities, or when clinicians require a more comprehensive assessment of coagulation status to guide clinical decision-making. Upon activation, the instrument continuously recorded the TEG curve in real time and automatically derived key parameters based on changes in the viscoelastic properties of the blood. These parameters included reaction time (R), coagulation time (K), alpha angle (α-angle), maximum amplitude (MA), and 30-minute clot lysis percentage (LY30). The definitions of TEG parameters were as follows:^19,20^

1. **R time:** Duration from test start to initial fibrin formation, indicated by a TEG amplitude of 2 mm.
2. **K time:** Time interval from the end of R to when the clot reaches a strength corresponding to 20 mm amplitude.
3. **α-angle:** The angle formed between the tangent of the TEG curve’s rising segment and the baseline, indicating the rate of clot strengthening.
4. **MA:** The maximum amplitude of the TEG curve, representing peak clot strength and stability
5. **LY30:** Percentage reduction in clot amplitude measured 30 minutes after MA, reflecting fibrinolytic activity.

Two independent investigators extracted data from the electronic medical records. Inconsistencies occurred in less than 5% of cases, primarily involving the classification of AF patterns due to ambiguous clinical notes and the verification of pre-admission medication history. These were resolved by a third reviewer through a detailed review of the patient’s complete medical records. All identifiable patient information was removed to anonymize data before analysis.

### Sample size and post-hoc power analysis

Given the retrospective nature of this predictive model study, no formal prospective sample size calculation was performed. Methodological rigor was maintained by adhering to the minimum of 10 events per predictor (EPV) for stable model estimation, supplemented by post-hoc power analysis to confirm sample size adequacy. The final model identified three independent predictors for PE in patients with AF, with 41 PE events among 271 enrolled subjects, yielding an EPV of 13.7—exceeding the recommended threshold of 10 and supporting reliable parameter estimation. Post-hoc power analysis, conducted using the pROC package in R, was based on the observed AUC of 0.878 and a sample size of 271. At a two-sided α level of 0.05, the statistical power exceeded 90% to detect an AUC significantly greater than 0.70 (the null hypothesis), indicating sufficient sample size to identify the model’s significant predictive performance. Furthermore, internal validation using 1000 bootstrap resamples produced a narrow optimism-corrected AUC confidence interval (0.804–0.916), reinforcing model robustness and the appropriateness of the sample size.

### Statistical analysis

All statistical analyses were performed using R software (version 4.3.0) and SPSS (version 26.0). The normality of data distribution was assessed using the Kolmogorov-Smirnov test. Descriptive statistics were used to summarize the data. Continuous variables with normal distribution were presented as mean ± standard deviation, while non-normally distributed data were expressed as median with interquartile range (IQR). Categorical variables were described as frequencies and percentages. Between-group comparisons were conducted using the independent samples t-test or Mann–Whitney U test, as appropriate. Chi-square test was used for categorical variables. Backward stepwise multivariate logistic regression analysis was employed to identify independent predictors of PE, and a nomogram model was subsequently constructed based on the final regression model. The discriminatory ability of the model was evaluated using the ROC curve and the AUC, where a higher AUC indicates better discrimination. DeLong’s test was used to compare AUCs between different models or subgroups to assess the statistical significance of discrimination differences. Model calibration was assessed by the Hosmer–Lemeshow goodness-of-fit test and calibration curves, examining the agreement between predicted and observed outcomes. Clinical utility was evaluated using DCA, which estimates net clinical benefit across a range of threshold probabilities. To assess model robustness and internal validity, bootstrap resampling (1,000 iterations) was performed. The average AUC of the validation sets was calculated, and a ROC curve was generated to further evaluate the model’s generalizability. Furthermore, a sensitivity analysis was conducted to assess the robustness of the findings by incorporating clinically relevant variables identified in univariate analysis into the multivariate logistic regression model. A two-sided P value < 0.05 was considered statistically significant.

## Results

### Baseline characteristics of study population

A total of 271 hospitalized patients with AF were enrolled, of whom 41 (15.13%) had concomitant PE (Table 1). Baseline demographic characteristics, including age, sex, BMI, and length of hospital stay, were comparable between the two groups. Similarly, AF type, comorbid conditions, and thromboembolic risk (median CHA_2_DS_2_-VASc score: 4.00) did not differ significantly. Echocardiographic assessment showed that patients with PE had a significantly lower LVEF compared with those without PE (60.20 ± 8.77% vs. 63.91 ± 8.81%, p = 0.013), whereas other echocardiographic parameters were comparable between groups. Notably, hs-CRP levels were markedly higher in patients with PE (median 14.40 vs. 2.92 mg/L, p < 0.001), whereas other laboratory indices, including renal function, lipid profiles, and platelet counts, were similar across groups.

**Table 1:** – Comparison of Baseline Characteristics between Pulmonary Embolism Group and Non-Pulmonary Embolism Group

Variables	Total (n = 271)	Non-PE (n = 230)	PE (n = 41)	p
**Age (years)**	76.48 ± 10.20	76.00 ± 10.21	77.51±8.64	0.320
**Gender, n (%)**				0.282
Male	153 (56.46)	133 (57.83)	20 (48.78)	
Female	118 (43.54)	97 (42.17)	21 (51.22)	
**BMI (kg/m^2^)**	22.86 (20.28. 25.28)	23.00 (20.28. 25.28)	22.39 (20.41. 25.23)	0.618
**Hospital stays (days)**	7.81 (5.14. 12.92)	7.11 (5.12. 12.92)	8.79 (5.21. 12.16)	0.579
**AF Pattern, n (%)**				0.351
Paroxysmal AF	70 (25.83)	57 (24.78)	13 (31.71)	
Non-Paroxysmal AF	201 (74.17)	173 (75.22)	28 (68.29)	
**Medical history**				
**Hypertension, n (%)**				0.634
No	88 (32.47)	76 (33.04)	12 (29.27)	
Yes	183 (67.53)	154 (66.96)	29 (70.73)	
**Diabetes mellitus, n (%)**				0.233
No	211 (77.86)	182 (79.13)	29 (70.73)	
Yes	60 (22.14)	48 (20.87)	12 (29.27)	
**Coronary artery disease, n (%)**				0.386
No	122 (45.02)	101 (43.91)	21 (51.22)	
Yes	149 (54.98)	129 (56.09)	20 (48.78)	
**Thrombocytopenia, n (%)**				0.819
No	228 (84.13)	194 (84.35)	34 (82.93)	
Yes	43 (15.87)	36 (15.65)	7 (17.07)	
**COPD, n (%)**				0.907
No	223 (82.29)	189 (82.17)	34 (82.93)	
Yes	48 (17.71)	41 (17.83)	7 (17.07)	
**Heart failure, n (%)**				0.782
No	235 (86.72)	200 (86.96)	35 (85.37)	
Yes	36 (13.28)	30 (13.04)	6 (14.63)	
**Cerebral infarction, n (%)**				0.296
No	179 (66.05)	149 (64.78)	30 (73.17)	
Yes	92 (33.95)	81 (35.22)	11 (26.83)	
**Malignant tumor, n (%)**				0.587
No	226 (83.39)	193 (83.91)	33 (80.49)	
Yes	45 (16.61)	37 (16.09)	8 (19.51)	
**CHA_**2**_DS_**2**_-VASc score**	4.00 (3.00. 6.00)	4.00 (3.00. 6.00)	4.00 (3.00. 5.00)	0.474
**Laboratory parameters**				
PLT (×10^9^/L)	166.00 (128.50. 212.00)	168.50 (129.00. 215.75)	157.00 (125.00. 196.00)	0.338
Hs-CRP (mg/L)	3.80 (1.19. 12.60)	2.92 (0.97. 9.96)	14.40 (3.50. 35.76)	<0.001
UA (μmol/L)	310.00 (236.00. 379.00)	309.00 (238.00. 369.00)	317.00 (189.00. 441.00)	0.642
Scr (μmol/L)	78.00 (64.00. 103.00)	79.50 (65.00. 99.25)	74.00 (61.00. 124.00)	0.923
eGFR (mL/min/1.73m^2^)	77.26 (57.97. 97.03)	76.98 (58.83. 96.21)	79.67 (45.00. 109.28)	0.818
TG (mmol/L)	1.12 (0.80. 1.60)	1.11 (0.79. 1.60)	1.17 (0.83. 1.60)	0.600
TC (mmol/L)	3.73 (3.11. 4.52)	3.69 (3.11. 4.56)	3.82 (3.09. 4.39)	0.787
HDL-C (mmol/L)	1.18 (0.97. 1.39)	1.20 (0.98. 1.40)	1.07 (0.85. 1.28)	0.099
LDL-C (mmol/L)	2.16 (1.70. 2.80)	2.15 (1.68. 2.76)	2.25 (1.87. 2.96)	0.319
**Echocardiographic parameters**				
Aortic root diameter	27.18 ± 4.09	27.06 ± 4.25	27.94 ± 2.87	0.152
LAD (mm)	39.71 ± 8.04	39.57 ± 7.92	40.55 ± 8.83	0.530
IVS (mm)	8.94 ± 1.38	8.99 ± 1.41	8.65 ± 1.14	0.191
LVPW (mm)	8.96 ± 1.34	9.02 ± 1.32	8.65 ± 1.38	0.153
LVEDD (mm)	47.50 ± 6.72	47.64 ± 6.62	46.71 ± 7.38	0.478
LVESD (mm)	30.54 ± 6.50	30.63 ± 6.55	30.00 ± 6.26	0.620
LVEF (%)	63.35 ± 8.89	63.91 ± 8.81	60.20 ± 8.77	0.013

Thrombocytopenia was defined as a platelet count < 100 × 109/L. PE: pulmonary embolism; BMI: body mass index; COPD: chronic obstructive pulmonary disease; LAD: left atrial diameter; IVS: interventricular septal thickness; LVPW: left ventricular posterior wall thickness; LVEDD: left ventricular end-diastolic diameter; LVESD: left ventricular end-systolic diameter.

### Comparison of TEG parameters and coagulation markers between groups

As shown in Table 2, significant differences were found in TEG parameters and coagulation markers between the two groups. Patients in the PE group had significantly higher α-angle and MA compared to those in the non-PE group. Additionally, R time, K time, and LY30 were significantly different between groups. Levels of FIB and D-dimer were also markedly elevated in the PE group. In contrast, no significant differences were observed in PT, PT activity, INR, APTT, or TT between the groups.

**Table 2:** – Comparison of TEG parameters and coagulation indicators between PE and non-PE embolism groups

Variables	Total (n = 271)	Non-PE (n = 230)	PE (n = 41)	p
PT (s)	15.12 ± 3.93	15.16 ± 3.97	14.88 ± 3.74	0.679
PT activity (%)	70.83 ± 19.24	70.71 ± 19.56	71.48 ± 17.59	0.815
INR	1.32 ± 0.40	1.32 ± 0.40	1.30 ± 0.40	0.759
APTT (s)	30.76 ± 6.37	30.96 ± 6.39	29.65 ± 6.22	0.228
TT (s)	16.06 ± 1.31	16.06 ± 1.24	16.05 ± 1.68	0.986
FIB (g/L)	3.39 (2.43. 4.75)	3.26 (2.39. 4.52)	4.77 (2.68. 6.62)	0.001
D-dimer (mg/L)	0.82 (0.54. 1.20)	0.74 (0.48. 1.06)	1.12 (0.79. 2.93)	<0.001
R time (min)	3.70 (2.90. 4.60)	3.80 (3.00. 4.70)	3.20 (2.60. 3.80)	0.001
K time (min)	1.20 (0.90. 1.60)	1.30 (1.00. 1.70)	1.10 (0.80. 1.30)	0.002
LY30 (%)	1.10 (0.10. 3.20)	1.20 (0.10. 3.60)	0.80 (0.00. 1.60)	0.026
α angle (°)	69.54 ± 5.72	69.14 ± 5.94	71.81 ± 3.57	<0.001
MA (mm)	56.73 ± 4.57	56.00 ± 4.00	60.81 ± 5.40	<0.001

TEG: thromboelastography; PT: prothrombin time; INR: international normalized ratio; APTT: activated partial thromboplastin time; TT: thrombin time; FIB: fibrinogen; MA: maximum amplitude; LY30: lysis at 30 minutes; α-angle: clot strengthening speed; R: reaction time; K: clot kinetics.

### Independent risk factors for PE in AF patients

Univariate logistic regression analysis identified R time, K time, α-angle, LY30, MA, FIB, and D-dimer as significant factors associated with PE in AF patients. Subsequent multivariate analysis using backward stepwise regression revealed that: MA, FIB, and D-dimer were independent risk factors for PE in AF patients. Other variables did not retain statistical significance in the multivariate model (Table 3).

**Table 3:** – Univariate and multivariate logistic regression analysis of risk factors for PE in AF Patients

Variables	Univariate Analysis		Multivariate Analysis	
	p	OR (95%CI)	p	OR (95%CI)
R time (min)	0.002	0.62 (0.46 ~ 0.84)		
K time (min)	0.010	0.32 (0.13 ~ 0.76)		
α angle (°)	0.007	1.10 (1.03 ~ 1.18)		
MA (mm)	<0.001	1.30 (1.18 ~ 1.43)	<0.001	1.37 (1.21 ~ 1.54)
LY30 (%)	0.005	0.72 (0.57 ~ 0.91)		
FIB (g/L)	<0.001	2.20 (1.57 ~ 3.09)	<0.001	2.30 (1.46 ~ 3.62)
D-dimer (mg/L)	<0.001	2.55 (1.78 ~ 3.65)	<0.001	2.81 (1.77 ~ 4.47)

OR: odds ratio; CI: confidence interval; MA: maximum amplitude; FIB: fibrinogen; LY30: lysis at 30 minutes.

### Nomogram construction for predicting PE in AF patients

Based on the results of multivariate logistic regression analysis, a nomogram was constructed to predict the risk of PE in patients with AF (Figure 2). The model incorporated three independent predictors: D-dimer, FIB, and TEG-MA. To utilize the nomogram clinically, the value of each predictor is first located on its respective variable axis. A vertical line is drawn upwards to the “Points” axis to determine the specific score for that variable. The sum of these individual scores constitutes the “Total Points,” which is then located on the Total Points axis. Finally, a vertical line is drawn downwards from the Total Points axis to the “Risk” axis to obtain the individual predicted probability.

**Figure 2:** – Nomogram to predict the probability of PE in patients with AF.

### Model performance and validation

To evaluate the discriminative ability of the nomogram, a ROC curve was plotted. The AUC was 0.878 (95% CI: 0.806–0.949), indicating excellent predictive performance (Figure 3A). At the optimal cut-off value of 0.204, the model yielded a sensitivity of 87.8% and a specificity of 78.0%. Compared with the individual predictors (MA, FIB, and D-dimer), the nomogram demonstrated a significantly higher AUC (Table 4), suggesting that combining multiple factors can substantially enhance predictive accuracy.

**Figure 3:** – Performance evaluation of the nomogram model for PE in AF patients. (A) ROC curve of the nomogram model; (B) Calibration curve of the nomogram model; (C) DCA curve; (D) ROC curve after internal validation using the Bootstrap method.

**Table 4:** – Comparison of predictive performance between the nomogram model and individual biomarkers

Variables	AUC (95%CI)	Sensitivity	Specificity	Cut off	Delong-test
Nomogram	0.878 (0.806-0.949)	87.8%	78.0%	0.204	reference
TEG-MA (mm)	0.771 (0.680-0.862)	72.6%	73.2%	58.2	<0.001
FIB (g/L)	0.741 (0.669-0.813)	70.0%	70.7%	3.199	<0.001
D-dimer (mg/L)	0.742 (0.662-0.821)	95.2%	41.5%	2.011	<0.001

AUC: area under the curve; CI: confidence interval; MA: maximum amplitude; FIB: fibrinogen.

In terms of calibration, the calibration plot (Figure 3B) demonstrated good agreement between predicted probabilities and actual observed outcomes. The Hosmer–Lemeshow goodness-of-fit test yielded a p-value of 0.965 (>0.05), further supporting the model’s good fit in the training cohort.

To assess the clinical utility of the nomogram, DCA was conducted. The DCA curve (Figure 3C) showed that within a threshold probability range of 0.2 to 0.8, the model provided greater net benefit than either the “treat-all” or “treat-none” strategies. The maximum net benefit was observed at a threshold of 0.3, suggesting that the model could help avoid approximately 120 unnecessary interventions per 1,000 patients—demonstrating strong clinical applicability.

In addition, to validate the model’s robustness, internal validation was performed using bootstrapping with 1,000 resamples. The average AUC obtained was 0.860 (95% CI: 0.804–0.916), indicating good generalizability and stability of the model (Figure 3D).

### Sensitivity analysis and model comparison

To further evaluate the robustness of our findings and assess the potential added value of clinical characteristics, we performed a sensitivity analysis. In this analysis, clinical variables with a univariate p<0.05 (specifically, Hs-CRP and LVEF) were introduced into the multivariate logistic regression model alongside the coagulation parameters. In this extended model, LVEF was identified as a significant independent predictor (OR: 0.95, p=0.027), while Hs-CRP did not retain significance. The extended model (combining MA, Fibrinogen, D-dimer, and LVEF) yielded an AUC of 0.893 (95% CI: 0.830-0.957). However, the DeLong test demonstrated that this improvement was not statistically significant compared to our original three-variable model (AUC 0.878 vs. 0.893, p=0.057). Therefore, considering the principle of model parsimony and clinical utility - where the original model requires only a single blood draw without the need for immediate echocardiography - we retained the coagulation-based model (MA, Fibrinogen, D-dimer) for the construction of the final nomogram.

## Discussion

In this study, we developed a nomogram model to predict the risk of PE in hospitalized patients with AF by integrating TEG parameters with conventional coagulation indices. The results demonstrated that MA, FIB, and D-dimer levels were independent risk factors for PE, and the constructed model exhibited good predictive performance. Given its non-invasive nature, ease of use, and suitability for bedside application, this model provides a novel tool for the early identification of high-risk PE among patients with AF.

The prevalence of PE in our study was 15.1%, which is notably higher than that reported in general AF populations. For instance, a multicenter study in China by Bai et al.^21^ reported an in-hospital PE prevalence of 1.2% among all AF patients. Similarly, large-scale population-based studies by Friberg et al.^22^ and Hald et al.^6^ observed much lower incidence rates, ranging from 2.9 to 18.5 per 1,000 person-years. This discrepancy in our study is primarily attributable to the specific inclusion criteria used: we restricted our analysis to patients who underwent CTPA, a diagnostic test typically reserved for individuals with a high clinical suspicion of PE (e.g., unexplained dyspnea, hypoxemia, or elevated D-dimer). Consequently, our study population represents a ‘high-risk suspected PE’ cohort rather than a general AF screening population. This distinction is crucial, as our model is specifically designed to assist clinicians in ruling in or ruling out PE within this challenging group of suspected patients.

Elevated D-dimer was identified as an independent predictor of PE in patients with AF, aligning with prior studies.^23,24^ As a fibrin degradation product, D-dimer signifies active coagulation and fibrinolysis.^25^ Although D-dimer specificity decreases with age, our study demonstrates that integrating it with TEG-MA and fibrinogen significantly enhances diagnostic accuracy compared to D-dimer alone. This multi-parameter approach effectively mitigates the false-positive rate often seen in elderly populations. From a pathophysiological perspective, the link between AF and PE is primarily indirect.^6^ While paradoxical embolism via an intracardiac shunt, such as a patent foramen ovale, represents a direct pathway, AF more commonly promotes a systemic hypercoagulable and pro-inflammatory state, impairs cardiac output, and causes venous stasis. These factors increase the risk of deep vein thrombosis and in-situ right heart thrombosis, which then serve as the primary sources for PE.^26-28^ Large cohort studies confirm that AF significantly elevates the risk of both pulmonary embolism and ischemic stroke, particularly in the first 6 months after diagnosis.^6^ Furthermore, AF is associated with pulmonary hypertension, which may worsen right heart function and promote thrombogenesis, creating a vicious cycle.^26^ Thus, the elevated D-dimer, fibrinogen, and MA in our model likely reflect this systemic thrombotic tendency. Importantly, since our study excluded patients on anticoagulation, these parameters provide a direct assessment of the intrinsic hypercoagulable state in AF patients, identifying AF patients at higher risk for PE principally via the venous thromboembolism pathway.

FIB is a key substrate in the coagulation cascade, and its elevated levels indicate a hypercoagulable state and contribute to both thrombus formation and stabilization.^29^ Studies have shown a significant association between elevated FIB levels and the risk of venous thromboembolism.^30,31^ AF itself represents a chronic low-grade inflammatory condition, and previous evidence has demonstrated persistently elevated levels of inflammatory markers such as C-reactive protein and interleukin-6. These inflammatory mediators can induce hepatic synthesis of FIB and coagulation factors, thereby enhancing the prothrombotic milieu.^32,33^ Furthermore, increased FIB levels can raise blood viscosity and promote erythrocyte aggregation, which exacerbates sluggish blood flow and creates a favorable environment for thrombogenesis.^34^ These mechanisms collectively predispose AF patients to PE in the setting of elevated fibrinogen levels.

In addition, we observed that patients with PE had significantly higher hs-CRP levels than those without PE. This finding further supports the potential role of systemic inflammation in the prothrombotic state of AF patients with PE. Elevated hs-CRP may reflect enhanced inflammatory activity associated with increased fibrinogen expression in AF, as well as endothelial and platelet-related thromboinflammatory processes that may contribute to thrombus formation. Although hs-CRP was not retained as an independent predictor in the multivariate model after adjustment for coagulation parameters, its marked elevation in the PE group underscores the close interplay between inflammation and coagulation in this high-risk population.^35,36^

MA is a critical TEG-derived parameter reflecting overall clot strength, determined by both platelet function and fibrin structure.^37,38^ In our study, elevated MA was significantly associated with AF-related PE, suggesting a pivotal role of platelet activation in thrombogenesis among these patients. Notably, although thrombocytopenia can theoretically lower MA, its prevalence was balanced between groups in our study (P=0.819). This indicates that the observed elevation of MA in the PE group reflects a genuine hypercoagulable state—likely driven by enhanced platelet function and increased fibrinogen—rather than a bias introduced by platelet counts. Thus, MA provides a functional marker of thrombotic potential, complementing the static information provided by platelet count. Prior research has shown that MA can predict thrombotic complications following orthopedic surgery and in patients with gastrointestinal bleeding.^39,40^ In AF, hemodynamic disturbances within the atria often result in local blood stasis and turbulence, which promote fibrin formation and platelet aggregation, leading to denser and more stable thrombi.^29,41^ Once dislodged, these thrombi may travel through the circulation and obstruct the pulmonary arteries, resulting in PE.^9,42^ As a sensitive indicator of blood viscoelasticity, MA may serve as a valuable adjunct for early PE risk stratification in the AF population.

We further contextualized our TEG-based nomogram against existing predictive tools. Traditional clinical scores (e.g., Wells, Geneva) exhibit limited specificity in elderly AF patients due to symptom overlap. Recent models have explored different approaches: a clinical nomogram by Ling et al.^7^ achieved an AUC of 0.721, while an AI-ECG model by Valente Silva et al.^43^ showed high specificity (100%) but lower sensitivity (50%) with an AUC of 0.75. Epidemiological studies have linked AF severity (via CHA_2_DS_2_-VASc score) to VTE risk,^32,44^ yet this score itself demonstrated modest predictive utility for PE (AUC 0.62). In contrast, our model integrates the dynamic functional marker TEG-MA, yielding superior discrimination (AUC 0.878) with a balanced sensitivity of 87.8% and specificity of 78.0%. This suggests that assessing real-time coagulation function offers enhanced predictive value within this high-risk cohort.

The cost-effectiveness of incorporating TEG into AF management requires consideration. Although TEG is more costly than routine coagulation tests (e.g., PT, APTT), it remains substantially less expensive than CTPA. Blanket application to all AF outpatients may not be economically feasible. However, our model targets a defined, high-risk inpatient subgroup with clinical suspicion of PE, where the disease prevalence in our study was 15.1%. In this specific context, employing our nomogram for initial risk stratification could be cost-effective. By accurately identifying patients at highest risk, it may guide selective use of confirmatory CTPA, thereby reducing unnecessary scans, minimizing radiation/contrast exposure, and optimizing resource allocation compared to a universal imaging strategy.^45,46^

### Strengths and limitations

This study offers several clinical advantages. First, TEG provides a dynamic, reproducible, and bedside-accessible assessment of coagulation, complementing traditional static coagulation markers. Second, this is the first study, to our knowledge, to construct a PE risk prediction model specifically for the AF population based on MA, FIB, and D-dimer. The multi-parameter approach enhances model robustness and accuracy, particularly in scenarios with atypical symptoms or limited access to imaging modalities.

However, certain limitations should be acknowledged. First, this was a single-center retrospective study with a relatively small sample size. Patient identification based on ICD-10 coding may be subject to incomplete case capture and misclassification, potentially introducing selection bias. Second, it is important to note that the study was conducted at a specialized oncology hospital. Even with the strict exclusion of active malignancies, the patient population in this specific setting inherently presents greater frailty and a heavier burden of complex comorbidities compared to cohorts in general hospitals. Consequently, this specific setting may limit the generalizability of our findings to lower-risk populations. Third, due to the retrospective nature of data collection, several clinically important variables related to PE—including prior history of VTE, thrombophilia status, detailed signs and symptoms of PE, and right heart echocardiographic findings (including chamber size and function)—were not consistently available in the medical records and therefore could not be included in the analysis. Fourth, due to the limited number of PE events, we did not perform subgroup analyses for specific comorbidities such as heart failure or prior cerebral infarction. Future studies with larger cohorts are recommended to further explore the specific risk profiles of these subgroups. Fifth, the model lacks external validation, and its applicability across diverse populations remains uncertain. Finally, long-term follow-up data were unavailable, precluding the assessment of long-term predictive value. Future research should adopt a prospective, multicenter design with larger cohorts, incorporate additional biomarkers, and explore model utility in broader thromboembolic conditions.

## Conclusion

In summary, this study successfully developed a nomogram model to predict pulmonary embolism in patients with atrial fibrillation, based on D-dimer, fibrinogen, and MA. The model demonstrated satisfactory predictive performance and clinical applicability, offering a valuable tool for early identification of high-risk patients and formulation of individualized treatment strategies. Future multicenter, large-scale prospective studies are warranted to further validate and refine this predictive model.
